# ATF3 Regulates the Expression of AChE During Stress

**DOI:** 10.3389/fnmol.2018.00088

**Published:** 2018-04-06

**Authors:** Ronit Heinrich, Rivka Hertz, Esther Zemel, Irit Mann, Liat Brenner, Amir Massarweh, Shai Berlin, Ido Perlman

**Affiliations:** Department of Neuroscience, Ruth & Bruce Rappaport Faculty of Medicine, Technion-Israel Institute of Technology and The Rappaport Institute, Haifa, Israel

**Keywords:** retina, ATF3, AChE, degeneration, photic stress

## Abstract

Acetylcholinesterase (AChE) expresses in non-cholinergic cells, but its role(s) there remain unknown. We have previously attributed a pro-apoptotic role for AChE in stressed retinal photoreceptors, though by unknown mechanism. Here, we examined its promoter only to find that it includes a binding sequence for the activating transcription factor 3 (ATF3); a prototypical mediator of apoptosis. This suggests that expression of AChE could be regulated by ATF3 in the retina. Indeed, ATF3 binds the AChE-promoter to down-regulate its expressions *in vitro*. Strikingly, retinas of “blinded” mice display hallmarks of apoptosis, almost exclusively in the outer nuclear layer (ONL); coinciding with elevated levels of AChE and absence of ATF3. A mirror image is observed in the inner nuclear layer (INL), namely prominent levels of ATF3 and lack of AChE as well as lack of apoptosis. We conclude that segregated patterns of expressions of ATF3 reflect its ability to repress apoptosis in different layers of the retina—a novel mechanism behind apoptosis.

## Introduction

The central nervous system (CNS), including the retina, responds with unique cellular and genetic mechanisms to traumatic episodes. These are aimed to combat further damage by recovery processes or apoptosis of select cellular populations, in order to protect neighboring cells and/or the remaining tissue. One such recognized stress-induced mechanism in the brain involves, surprisingly, acetylcholinesterase (AChE; Meshorer and Soreq, 2006; Toiber et al., 2008). In the adult mammalian retina, a subset of inner retinal neurons, amacrine and ganglion cells, are involved in cholinergic synaptic activity (Hutchins, 1987; Yasuhara et al., 2003). Therefore, it isn’t surprising to find AChE expression in the inner plexiform layer (INL) of the retina, where amacrine and ganglion-cells form synaptic interactions. Unexpectedly, mRNA of AChE has also been detected in cells that are not involved in cholinergic transmission, such as in photoreceptors of fetal, adult and aging human retinas (Broide et al., 1999). These observations strongly support other, non-cholinergic, role(s) for AChE.

The observation that AChE may exhibit additional roles, such as in mechanisms of apoptosis, does not come as a complete surprise, in hindsight, as the alternative splicing of its pre-mRNA produces three distinct AChE isoforms. Whereas all variants contain the globular core responsible for hydrolysis of acetylcholine (ACh), the different variants display very divergent expression patterns: the “synaptic” (S) protein is found in all cholinergic synapses, the “read-through” (R) protein is exclusively expressed in embryonic cells, cancer cells and in response to stress, whereas the “erythrocytic” (E) protein specifically expresses in erythrocytes (Kaufer et al., 1998; Grisaru et al., 1999; Meshorer and Soreq, 2006). Further distinction between the variants is obtained by modifications in the enzyme’s amino-terminus (NT); in normal or extended form, denoted N-AChE (Meshorer et al., 2004).

We have recently described a novel non-cholinergic role played by AChE, involving the induction of apoptosis in retinal photoreceptors; following photic-stress *in vivo* (Kehat et al., 2007), or following hyperglycemic-stress of retinoblastoma cells, *in vitro* (Masha’our et al., 2012). These studies demonstrate that cellular stress can upregulate the expression of AChE; necessary for the induction of programmed cell death. However, the molecular mechanism behind this phenomenon and the exact enzymatic variant remain unknown. Curiously, the AChE-promoter, known to generate all the different variants of AChE, contains the consensus binding site “TGACGTCA” (Hai and Hartman, 2001) for members of the cyclic-AMP responsive element binding proteins. This suggests that AChE expression levels may be regulated by members belonging to the (activating-transcription-factor/cyclic-AMP-responsive-element-binding protein, ATF/CREB) superfamily. In support, AChE has been shown to be regulated by CREB in avian myotubes (Choi et al., 2001).

Of the various members, the activating transcription factor 3 (ATF3) is the best studied factor with regard to apoptosis in the CNS (Hunt et al., 2012). The ATF3 gene encodes a transcription factor displaying both pro- and anti-apoptotic effects (Nawa et al., 2002; Nobori et al., 2002). For instance, ectopic expression of ATF3, in cardiac myocytes, inhibits Doxorubicin-induced apoptosis, indicating a protective (anti-apoptotic) role of ATF3 (Nobori et al., 2002), whereas overexpression of ATF3 in cultured HeLa cells enhances etoposide (or camptothecin)-induced apoptosis, thereby displaying a strong pro-apoptotic role (Mashima et al., 2001). Despite the extensive body of work regarding ATF3 and apoptosis in various cell types, it is surprising to find that there are far less, or no data at all, regarding ATF3’s role in the retina. We, therefore, sought to examine whether ATF3 plays a role in the AChE-induced apoptosis in the retina. More precisely, we hypothesized that the expression of AChE is dependent upon ATF3’s activity or expression. Therefore, we set-out to scrutinize potential interactions between ATF3 and the AChE-promoter, *in vitro* and *in vivo*, in the retina of albino mice undergoing photic-stress.

## Materials and Methods

### Animals

Experiments were performed on male albino (Balb/C) mice, weighing 15–20 g; housed for 2 weeks in darkness, in ventilated light-tight chambers located in a separate dark room with free access to water and food. Cage cleaning and food supply was done under dim red illumination. Following the 2-weeks darkness period, mice were exposed to photic-stress for 0-, 2-, 10- or 24-h. Exposure to light for 0-h served as a control for the effects of 2-weeks of darkness on the albino mouse retina.

It is important to note that the rational of housing the mice for 2 weeks in complete darkness, prior to light exposure, was based on previous studies showing that the retina becomes highly susceptible to light damage after dark rearing (Noell and Albrecht, 1971; Hagins et al., 1989; Organisciak et al., 1998; Okawa et al., 2008). Our own experience shows that 2 weeks of darkness is a sufficient amount of time to produce a severe, and highly reproducible (consistent), light-induced retinal damage.

This study was carried out in strict accordance with the Guide for the Care and Use of Laboratory Animals of the National Institutes of Health. The protocol was approved by the Committee on the Ethics of Animal Experiments of the Technion (Permit Number: IL-031-02-2010).

### Cell Lines

Some of the experiments were performed on pheochromocytoma (PC12) cell line derived from rat adrenal medulla, on HEK293gp and on mouse embryonic fibroblasts (MEFs), generated by Prof. A. Aronheim (Technion, Israel) from C57/Bl ATF3KO mice (Darlyuk-Saadon et al., 2012). Cells were cultured in DMEM-growth medium supplemented with 10% FBS, 1% antibiotics and 1% Glutamine (Beit-Haemek, Israel). Plates were kept in a 37°C incubator at 5% CO_2_, and cell density was monitored once a week.

### Light Damage and Experimental Protocol

Mice were placed in ventilated light-exposure chambers (white light, 2600 lux), for a period of 2, 10 or 24-h. During light exposure, each mouse was housed separately in a clear plastic cage that was placed underneath a brtight fluorescent light-source. Room temperature, during light-exposure, was kept at 24°C.

Following light exposure, the extent of functional retinal damage was assessed from the dark-adapted electroretinogram (ERG). Then, both eyes from each mouse were enucleated: one was fixed for 1-h in PBS (pH = 7.4) containing 4% paraformaldehyde for immunocytochemistry or TUNEL assay. The retina from the other eye was removed, snap-frozen in liquid nitrogen and stored in −80°C for Real-Time PCR and chromatin immunoprecipitation (CHIP) analysis.

### Anesthesia

For ERG recording, mice were anesthetized by an intramuscular injection of a mixture containing ketamine hydrochloride (3.3 mg/kg), acepromazine maleate (6.66 μg/kg) and xylazine (2 μg/kg), at a dose of 0.5 ml/kg body weight. Three pain reflexes were checked to estimate the depth of anesthesia; the leg, tail and corneal reflexes. Preparation for ERG recording started only after the animal stopped responding to all three stimuli. Following ERG recording, additional dose of the anesthetic mixture was given in order to euthanize the mouse prior to eye enucleation.

### Electroretinogram (ERG)

Mice were kept over-night in darkness for complete dark adaptation. Preparations for ERG recording were done under dim red light. The pupils were fully dilated with cyclopentolate hydrochloride 1%, and topical anesthesia (Localin) was applied to the cornea to prevent any potential discomfort. A heating pad was used to maintain normal body temperature. The ERG was recorded with a corneal electrode, using a drop of methylcellulose (Cellospan) to maintain corneal hydration, and to ensure electrical contact. Reference and ground electrodes, made of stainless steel surgical needles, were inserted through the ear (reference electrode), and under the skin in the back (ground electrode).

Light stimuli were obtained from a Ganzfeld light source (LKC Technologies, Gaithersburg, MD, USA) with a maximum strength of 5.76 cd-s/m^2^. The strength of the light stimulus was attenuated manually by a set of neutral-density filters covering a range of 4 log units. Differential amplifiers (Grass Medical Instruments, Quincy, MA, USA) were used to amplify (10,000×) and filter (0.3–300 Hz) the ERG signals. The outputs of the amplifiers were digitized at a rate of 1 KHz by a computer equipped with a data acquisition board (LabVIEW 4.1 National Instruments, Austin, TX, USA). Six responses to six identical stimuli, applied at 10-s intervals, were averaged to improve signal/noise ratio. Data analysis was based on amplitude measurements of the ERG waves. The a-wave was measured from the baseline to its trough, and the b-wave was measured from the trough of the a-wave to the peak of the b-wave (e.g., Figure 3C, inset). Since the ERG responses were severly reduced by the light exposure, we used amplitudes measured with the brightest test flash (5.76 cd-s/m^2^) to define the maximum response amplitude of the dark-adapted ERG a- and b-waves.

**Figure 1:**
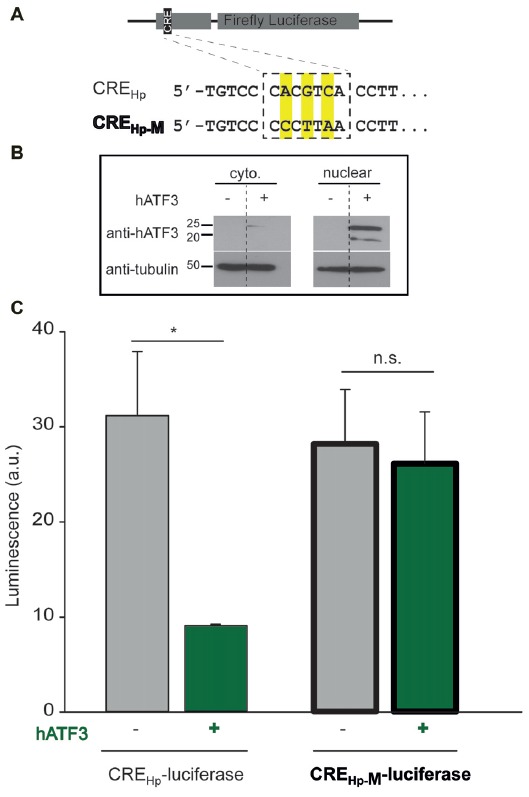
Acetylcholinesterase (AChE) promoter activity. **(A)** Pheochromocytoma (PC12) were transiently transfected with an empty vector lacking (-hATF3) or including (+hATF3) the human activating transcription factor 3 (ATF3) insert (pCan-empty and pCan-hATF3, respectively) together with biolouminescent reporter plasmids containing Renilla Luciferase (pGL3-RLU). The Renilla Luciferase was under the control of two different human AChE-promoter (Hp) that included the consensus cyclic AMP-responsive element (CRE) sequence (CRE_Hp_) or a mutated CRE sequence (CRE_Hp-M_). A Renilla Luciferase plasmid (pRLO-RLU) was used for normalizing expression (see “Materials and Methods” section). 48-h post transfection, luciferase activity was monitored using a luminometer. **(B)** Western blots of cytoplasmic (left, top lane) and nuclear (right, top lane) extracts from PC12 transiently overexpressing hATF3 (pCan-hATF3 denoted +) or empty vector; pCan (denoted-). Tubulin was used for loading control (bottom lanes). Sizes of protein are shown in kD. **(C)** Note that luciferase activity (i.e., AChE expression) is significantly larger in PC12 cells that do not overexpress ATF3 (gray bars) or that contain a mutated promoter (bold bars). Each mammalian expression plasmid (excluding the reporter ones), was verified for its protein expression by Western blot (data not shown). Values are presented as mean ± SEM (*N* = 3); *T*-test, *p < 0.05; n.s., non-significant.

**Figure 2:**
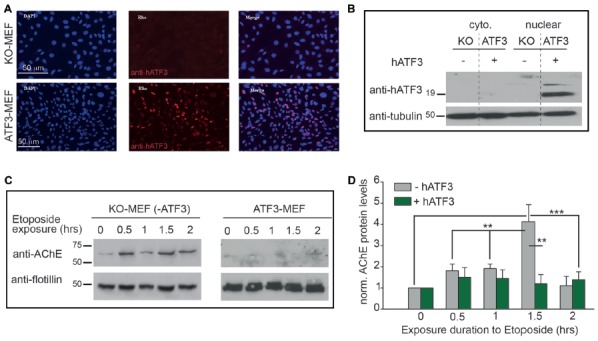
Interactions between ATF3 and AChE in mouse embryonic fibroblasts (MEFs) derived from ATF3 *knockout* (KO) mice, following exposure to apoptotic stress. **(A)** Stable expression of ATF3. MEF cells taken from ATF3-KO mice, stably transfected with an empty vector- pBabe (KO-MEF, top lane), do not show any expression of ATF3; assessed by immunofluorescence (middle lane, red). A stable MEF-cell line stably overexpressing human ATF3 (hATF3; ATF3-MEF, bottom lane) was generated by using pBabe-ATF3 viral transfection (see “Materials and Methods” section). ATF3-MEF cells show strong expression of ATF3 (red) in cell nuclei (blue, DAPI-staininig). **(B)** ATF3 expresses only in nuclear extracts of stable-cell lines overexpressing ATF3. ATF3 expresses only in the nuclear extract of MEF cells stably expressing hATF3 (lane 4, ATF3) and not in the nuclear extract of KO-MEF (lane 3, KO), neither in the cytoplasmic extracts of both cells lines (lanes 1 and 2). Tubulin was used for loading control. **(C)** Blots of membrane extracts from KO-MEF (left) and ATF3-MEF (right), following different exposure times to etoposide (40 μg/ml). Anti-Flottilin served as loading control. AChE appears as a diffusive band between 50 kDa and 75 kDa (molecular weight markers indicated on left of blot). **(D)** Summary of three independent experiments as shown in **(C)**. Values are presented as means ± SEM (*N* = 3); one way ANOVA, **p < 0.01; ***p < 0.001.

**Figure 3:**
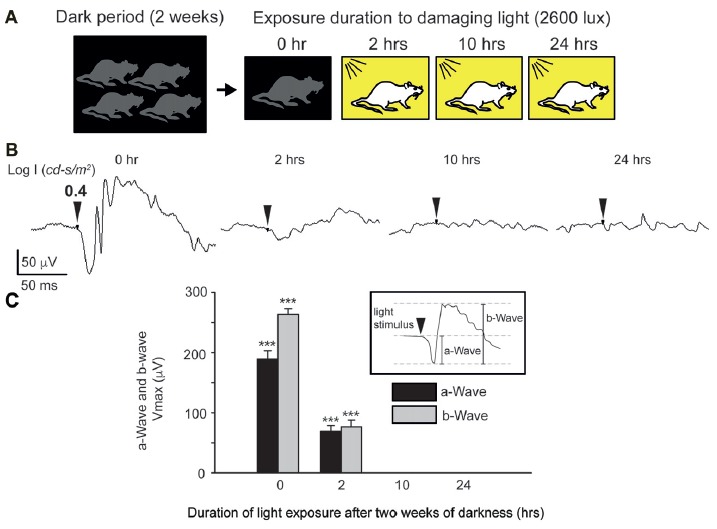
Photic stress of albino mice. **(A)** Protocol for inducing photic Stress. Albino mice are kept for 2 weeks in darkness before being exposed to bright damaging light. **(B)** Dark-adapted electroretinogram (ERG) responses, elicited by bright light stimuli (LogI = 0.76 cd-s/m^2^), recorded following 0-, 2-, 10-, and 24-h exposure to bright damaging light. Each trace describes the ERG of one eye of one mouse. For each mouse, the maximal amplitudes of the a-wave and b-wave were determined from the ERG responses that were elicited by the brightest light stimuli. Mean ± SEM Vmax values of dark-adapted a- and b-waves of the ERG of albino mice exposed to bright damaging light for different duration, summarized in **(C)**. In all three groups of mice (*N* = 12 in each group), exposure to light for 2-h, 10-h and 24-h, the maximal a-wave amplitude and maximal b-wave amplitudes were significantly (***p < 0.001) smaller than the corresponding values in the control mice that were rained for 2 weeks in complete darkness, but were not exposed to bright damaging light (exposure time 0-h).

ERG Vmax values were compared using ANOVA with Sigmaplot 11 software (Jandel Scientific, San Rafael, CA, USA). Data were considered statistically significant if *p* < 0.05.

### Immunohistochemistry

Fixed eyes were washed in PBS, then cryo-protected in 15% and in 20% sucrose for 1 h each, and then in 30% sucrose for overnight at 4°C. The eyecups were embedded in OCT, cryo-cut into 16-μm thick sections along the vertical meridian, and mounted on glass slides. Retina cryo-sections were exposed to antibody against ATF3 (provided by Prof. Ami Aronheim, Technion), or against AChE (sc-6432, Santa Cruz Biotechnology, Inc., Santa Cruz, CA, USA) at a dilution of 1:200 in assay buffer; 0.1% Triton and 3% FBS in PBS, for overnight at 4°C. The sections were washed in PBS and incubated with secondary antibodies (Jackson ImmunoResearch Laboratories, Inc., West Grove, PA, USA), conjugated to Rhodamine (donkey anti-rabbit for ATF3) or Cy3 (donkey-anti goat for AChE) at a dilution of 1:500 in assay buffer; 0.1% Triton and 3% FBS in PBS, for 1 h at room temperature, while prevented from exposure to light. The sections were washed in PBS, stained with DAPI (1 mg/ml stock solution) at a 1:1000 dilution in PBS for 5 min, and then washed three times in PBS, mounted and cover-slipped for viewing by confocal microscopy (LSM-700, Zeiss). Omitting primary antibody in the immunostaining procedures served as a negative control.

The sections were analyzed by 561 nm excitation wavelength, and later processed using IMARIS^®^ scientific 3D/4D image processing and analysis software. For quantification, 2000–10,000 cells were counted in each tested field, and the fractions of cells stained for AChE or ATF3 were calculated.

### TUNEL Assay

*In situ* cell death detection kit (Roche, Germany) was used for detection and quantification of apoptosis at the single cell level, based on labeling of DNA strand breaks. The assay was performed on cryo-sections in accordance with manufacturer instructions. The sections were analyzed by 488 nm excitation wavelength, and processed later using IMARIS^®^ scientific 3D/4D image processing and analysis software. For quantification, 2000–10,000 cells were counted in each tested field, and the fractions of apoptotic cells were calculated.

### Reporter Assay

We used the Trans IT-LT1 reagent (MIRUS, Atlanta, GA, USA) for transfection according to the manufacturer instructions. 24-h before transfection, PC12 cells were split in a 10 cm^2^ plate to reach ~70% confluency. Eight microgram of plasmid DNA (1 μg Renilla plasmid used for normalizing transfection efficiency, 1 μg reporter plasmid and 6 μg mammalian expression vectors) were added to the diluted *Trans*IT-LT1 reagent, by gentle pipetting and incubation for 30 min at room temperature. The *Trans*IT-LT1 reagent-DNA complex was added to the cells, and was incubated for 48 h in a 37°C incubator at 5% CO_2_. Then, cells were harvested and a reporter assay was performed, using the Dual-Luciferase Reporter Assay System kit (Promega, Madison, WI, USA) according to manufacturer instructions. pCan-myc-ATF3 mammalian expression plasmid (with a CMV promoter), excluding the reporter ones, was verified for its ATF3 protein expression levels by Western blot.

### Plasmids Source for Reporter Assay

pCan-myc-human ATF3 and Renilla luciferase (pRLO-RLU) plasmids—provided by Prof. A. Aronheim, Technion. pGL3-AChE-luciferase and pGL3-AChE_ΔCRE_-luciferase—provided by Prof. K. Tsim, Hong Kong University (Choi et al., 2001).

### Western Blot Analysis

Cells were lysed in a cytoplasmic lysis buffer (in mM: 10 HEPES, pH 7.9, 10 KCL, 1 EDTA, 1 DTT, 0.5 PMSF) and then in a nuclear lysis buffer (in mM: 20 HEPES, pH 7.9, 400 NaCl, 1 EDTA, 1 DTT, 0.5 PMSF) containing a protease inhibitor cocktail (Schreiber et al., 1989). Membranous extracts were prepared using the MBL kit (JM-K268-50, USA) according to manufacturer instructions. Proteins were then separated by 10%–12.5% SDS-PAGE and transferred onto a nitrocellulose membrane. Membranes were incubated in 3% non-fat dry milk in PBS for 1 h at RT, and subsequently probed with one of the primary antibodies for 16 h at 4°C. The primary antibodies used were rabbit polyclonal anti-ATF3 (provided by Prof. Ami Aronheim, Technion), rabbit polyclonal anti-AChE (sc-11409, Santa Cruz Biotechnology, USA). Mouse monoclonal anti-α-Tubulin (T6199, Sigma-Aldrich, St. Louis, MO, USA) was used for normalizing cytoplasmic protein loading and mouse monoclonal anti-Flotillin (sc-28320, Santa Cruz Biotechnology, USA) was used for normalizing membranous protein loading. Primary antibodies were detected using the HRP-conjugated Donkey-anti-rabbit or anti-mouse (Jackson ImmunoResearch Laboratories, USA) secondary antibodies. Densitometry analysis was done by TotalLab Quant software.

### Real Time PCR (RT-PCR)

Retinas were isolated and snap-frozen in liquid nitrogen and then stored in −80°C until RNA extraction. RNA extractions were done with TRI reagent (Sigma- Aldrich, USA) in accordance with manufacturer instructions. Samples were subjected to real-time PCR by using the following primers:

For mouse AChE-read-through isoform (R) intron4 (RefSeq: NM_009599.4):

- mAChE I4 forward: 5′-GAGCAGGGAATGCACAAG-3′
- mAChE I4 reverse (ordered): 5′-GGGGAGGTGGAGAAGAGAG-3′

For mouse AChE-S isoform exon6 (RefSeq: NM_009599.4):

- mAChE E6 forward: 5′-CTGAACCTGAAGCCCTTAGAG-3′
- mAChE E6 reverse (ordered): 5′-CCGCCTCGTCCAGAGTAT-3′

For mouse N-extended-AChE (according to Meshorer et al., 2004):

- mN-extended-AChE forward: 5′-GGACCCTTGTGATGACAGC-3′
- mN-extended-AChE reverse: 5′-GAATTAGCTCAAGCCCAC-3′

GAPDH (normalizing housekeeping gene) primers mix—forward and reverse were used after checking for the right normalizing gene using the geNorm kit (Primerdesign^®^, UK).

### Generation of Stable ATF3-Expressing Cells

MEFs derived from ATF3 knock-out (KO) mice (provided by Prof. A. Aronheim, Technion), were infected with replication-defective retroviruses to generate MEFs cell lines overexpressing ATF3. Retroviruses expressing ATF3, or empty vector as control were generated by transfection of pCLBabe-ATF3 or pCLBabe-vehicle plasmids derived from the Moloney murine leukemia virus that contains an SV40 promoter, which drives the drug resistance for puromycin and an LTR promoter which drives the transgene (provided by Prof. A. Aronheim, Technion), into a viral packaging cell line HEK293gp, expressing the *gag* and *pol* genes (Naviaux et al., 1996). The medium of transfected HEK293gp cells containing retroviruses was used to infect MEF cells. Infected cells were selected with puromycin (2.5 μg/ml), and pooled following 2 weeks of selection.

### Chromatin Immuno-Precipitation (ChIP)

ChIP from mice retinas was performed using the Enzymatic Chromatin IP (Agarose Beads) kit (Cell Signaling Technology, Danvers, MA, USA), according to manufacturer instructions. Briefly, retinas were cross-linked with 4% paraformaldehyde, and genomic DNA was extracted and fragmented to 150–900 bp by sonication. Immunoprecipitation was performed with one of the following: (1) A non-relevant normal rabbit IgG. (2) A positive control anti-Histone 3 (both provided with the kit). (3) A relevant anti-ATF3 polyclonal antibody (Santa Cruz Biotechnology, USA). Following extensive washes, cross-linking was reversed and DNA was extracted. The precipitated purified DNA was used as a template for real-time PCR reactions with:

1. Appropriate primers used for detection of the flanking CRE region in AChE promoter (located at -2.3kb to -2.1kb within the mouse AChE- promoter) were (RefSeq: NM_009599.4):
  - Forward: 5′-GGGAGAGCCTGTGTTTCTGT-3′
  - Reverser: 5′-GGCATCTGTAACCAGGACAAA-3′
2. Primers detecting a non-specific sequence located at the coding region of AChE- gene located between +4bp and +26bp within the AChE coding region, served as negative control were (RefSeq: NM_009599.4):
  - Forward: 5′-CTCCCTGGTATCCCCTGCAT-3′
  - Reverser: 5′-ATGCCCCTCAGCTGGCCCC-3′
3. Primers for RPL30 gene (intron 2) served as positive control (provided in the kit) for testing the immuno-precipitation of chromatin using Histone H3 antibody ensuring that ChIP experiments performed successfully.

### Statistics

Values are presented as mean ± SEM. For multiple group comparisons, statistical significance was assessed by one way ANOVA, and *Post hoc* analysis was performed using Tukey’s test. Two-group were compared by using the student *T-test* (Sigmaplot 11). Significance are indicted as *p < 0.05, **p < 0.01 and ***p < 0.001.

## Results

### ATF3 Interacts With the Promoter of AChE to Regulate Its Expression

To test whether ATF3 interacts with the AChE-promoter, we employed two different approaches. First, we tested whether ATF3 directly binds the AChE-promoter and, then, examined the functional consequence of this potential interaction. To test for the binding, we used a luciferase-reporter assay, commonly employed to study gene expression at the transcriptional level. To this end, we transfected a plasmid encoding a Renilla Luciferase under the control of the human AChE-promoter containing the CRE-consensus site (Figure 1A, top inset, CRE_Hp_), in the presence or absence of overexpressed human ATF3 (hATF3) in rat derived neuronal pheochromocytoma cells (PC12). We confirmed that transfection of PC12 with hATF3 (pCan-hATF3) yielded robust overexpression of the protein in the nuclear fraction of the cells (Figure 1B, nuclear; +) and further find that this overexpression exerted a 3.4 fold suppression in bioluminescence, compared to the basal AChE-promoter reporter activity in the absence of ATF3 (Figure 1C, left gray and green bars, respectively). To test for the specificity of this interaction, we subsequently used a mutated AChE-promoter plasmid containing three mutations within the CRE consensus site (Figure 1A, CRE_Hp-M_). The mutated CRE-site in the promoter completely abolished the ability of ATF3 to regulate the expression of the Luciferase (Figure 1C, right bold borders bars).

Next, to further scrutinize the interaction between ATF3 and the AChE-promoter, we sought to investigate whether ectopic expression ATF3 could interfere with the expression of AChE following cellular stress. For these experiments, we first used mouse embryoinic fibrobalsts (MEF) devoid of ATF3 (derived from C57/Black ATF3-KO mice; KO-MEF). From these cells, we further generated a stable MEF cell-line that overexpresses ATF3 (stable-MEF). Antibody-stainings against ATF3 confirmed that control MEF cells from ATF3-KO mice did not show any expression of ATF3 (Figure 2A, KO-MEF; upper panels). Conversely, the stable-line generated (stable-MEF) displayed strong expression of ATF3, expectedly, at cells’ nuclei (Figure 2A, lower panels, red nuclei). This was further validated by Western blot of cytoplasmic and nuclear extracts of control (KO-MEF) and stable-MEF cells (Figure 2B), where a strong cross-reacting band (corresponding to the 19 kDa ATF3 protein) was solely detected in the nuclear extract from the stable-MEF group (Figure 2B, lane 4), but not in its cytoplasmic extract (Figure 2B, lane 2). In contrast, neither the cytoplasmic nor the nuclear extracts from control KO-MEFs showed any ATF3 expression (Figure 2B, lanes 1 and 3, respectively). Thus, these confirm that the MEF-lines generated indeed lack or overexpress ATF3; at the right cellular location.

To induce stress onto the different MEF cell-lines, we employed etoposide (Kaufmann, 1989; Mashima et al., 2001); an anti-tumor drug known for its inhibition of topoisomerase II and an antineoplastic drug that couples DNA damage to apoptosis (Mizumoto et al., 1994). In control MEFs lacking ATF3 (KO-MEF), Western blots for AChE showed that the expression levels of the enzyme were robustly elevated following treatment with 40 μg/ml etoposide, as expected. We observed a four-fold increase in expression of AChE after 1.5 h, relative to control cells that were incubated only with carrier but without the drug (Figure 2C, KO-MEF, 0-h and summary in Figure 2D). Strikingly, AChE could not be detected in the stable-MEF overexpressing ATF3 after exposure to etoposide. This lack of expression was seen in cells that were exposed only to carrier (Figure 2C, stable-MEF, 0-h and summary in Figure 2D), or for varying durations with etoposide. These results demonstrate that ATF3 strongly down-regulates the expression of AChE, particularly during stress.

### AChE and ATF3 in a Mouse Model of Photic-Stress

We next sought to investigate the potential interactions between ATF3 and AChE, *in vivo*, following stress. To address this, we first evaluated a well established retinal-stress model involving exposure of rodents to bright and damaging light (Figure 3). Notably, this is a well-established model for photoreceptor degeneration, and is widely used to study pathological mechanisms, explicitly apoptosis, in photoreceptors (Stone et al., 1999). In particular, we have previsouly used this model to establish that AChE’s expression are increased in apoptotic photoreceptors in the retina of albino rats (Kehat et al., 2007). Thus, this model deemes highly suitable for testing our hypothesis of the interaction between AChE and ATF3, under stressful conditions leading to changes in AChE’s expression and to apoptosis *in vivo*.

To induce light-dependent retinal damage and apoptosis, we kept mice for 2 weeks in complete darkness before exposing them for varying durations to bright light, in order to produce different degrees of retinal damage (Figure 3A). This protocol yielded robust damage to the retina (“blinded”-retinas); assessed by ERG measurements (Figure 3B), with mice exhibiting a substantial functional damage to the retina following exposure as short as 2-h to bright light, though retinas still displayed a measurable ERG response (Figure 3B, 2-h). However, mice undergoing 10- or 24-h exposure to bright light showed a complete loss of ERG responses (Figure 3B, 10- and 24-h and summary in Figure 3C). Since we could not construct reliable response-log stimulus strength relationships in order to drive the maximal amplitude of the dark-adapted ERG a-wave and b-wave, we defined these parameters from the response to the brightest stimulus. Similar results were obtained from 12 mice belonging to each group of light exposure; 0-h, 2-h and 24-h. Careful analysis indicated significant reduction in Vmax of the a-wave and of the b-wave in the groups of mice that were exposed for 2, 10 or 24-h to bright light compared to the control group (no light exposure after dark adaptation, 0-h exposure). Together, these results indicate severe light-induced functional damage to the distal retina of albino mice following exposure to bright light.

**Figure 4:**
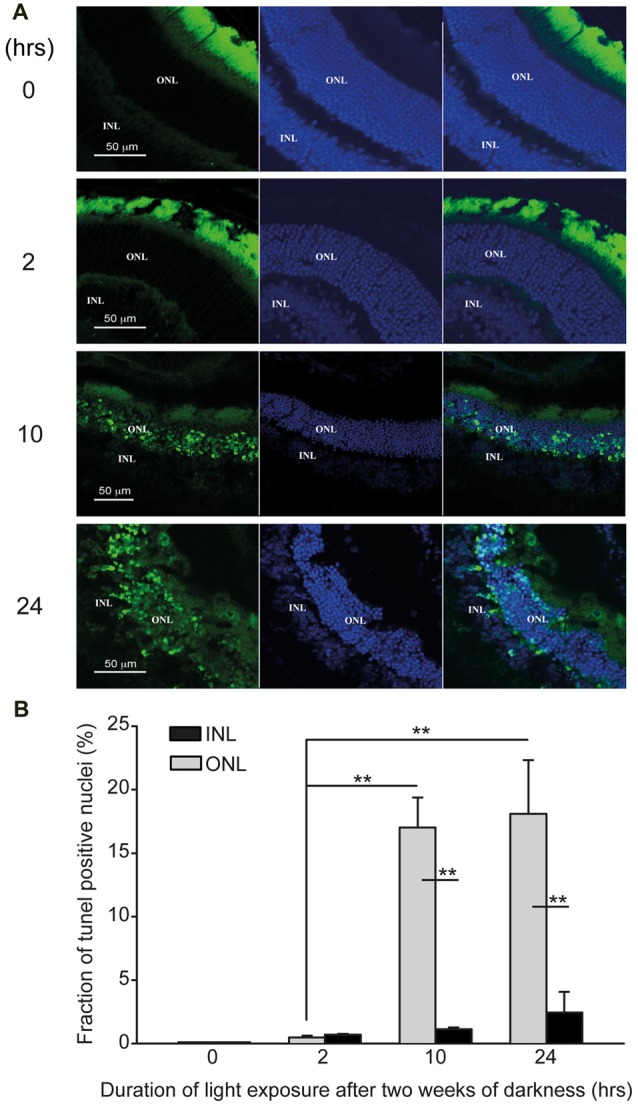
Photic-stress induces apoptosis in albino mice retina. **(A)** Representative retinal micrographs from mice, kept in complete darkness for 2 weeks and then exposed for 0- (A1), 2- (A2), 10- (A3), or 24-h (A4) to bright damaging light are shown (Blue-DAPI staining of cell nuclei, green-TUNEL positive cells). **(B)** Percentage of TUNEL positive cells in the outer nuclear layer (ONL) and inner nuclear layer (INL) of each retina was calculated using IMARIS^®^ scientific 3D/4D image processing and analysis *software*. Mean ± SEM, (*N* = 4, one mouse from each group from four independent experiments) show significant (**p < 0.01) larger percentage of TUNEL positive cells in the ONL compared to INL for mice exposed to light for 10-h or 24-h. Percentage of TUNEL positive cells in ONL of mice exposed for 2-h, 10-h or 24-h to damaging light was significantly (**p < 0.01) larger compared to control (0-h light exposure).

To assess structural damage and induction of apoptosis in “blinded”-retinas, we used the TUNEL assay on frozen sections of retinas from albino mice (Figure 4A). TUNEL-positive and DAPI-positive nuclei were counted in the outer nuclear layer and in the inner nuclear layer (ONL and INL, respectively), to determine the fraction of TUNEL-positive cells in each nuclear layer of the retinas (Figure 4B). In non-exposed, control retinas, no apoptotic nuclei were detected (Figure 4A, 0-h), whereas retinas exposed for 2-h to bright damaging light displayed a small percentage of apoptotic cells in the ONL (0.47 ± 0.1%), though none in the INL (Figure 4A, 2-h and Figure 4B). Nevertheless, apoptotic nuclei were highly abundant in mouse retinas undergoing longer exposures to bright damaging light (10-h: 17.02 ± 2.4%; 24 h: 18.09 ± 4.3%, respectively). These represent 36- and 38-fold increases, respectively, compared to mice exposed to only 2-h of light. Strikingly, apoptotic nuclei were almost exclusively localized to the ONL of the retina (Figure 4A, 10- and 24-h and Figure 4B). We observed a small trend of increase in the amount of apoptotic cells in the INL of “blinded”-retinas, though it did not reach significance (Figure 4B, black bars). The massive damage detected in the ONL strongly correlates with the deficits in the ERG responses, as the ONL includes the photoreceptor nuclei which, when non-damaged, give rise to the electrical responses detected by the ERG.

**Figure 5:**
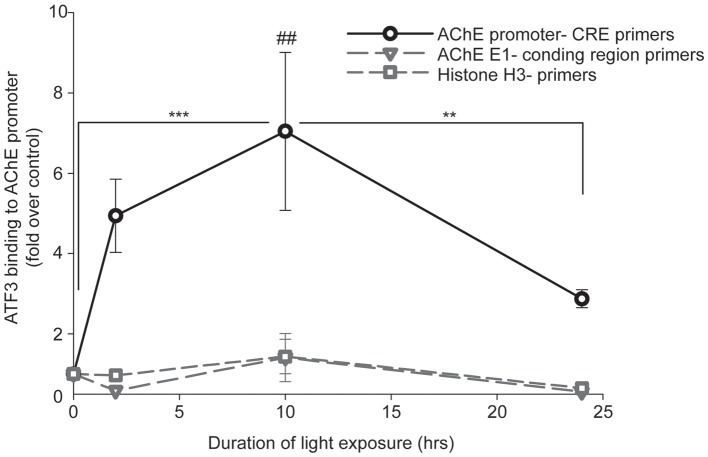
Association of ATF3 with AChE-promoter following light damage in albino mice tested by Chromatin immunoprecipitation (ChIP). Mice were kept in complete darkness for 2 weeks, and then exposed for 2-, 10- or 24-h to bright damaging light. At the end of light exposure, eyes were enucleated and retinas were isolated for ChIP assay as described in “Materials and Methods” section. Real-time PCR results from each light-exposure duration were calculated as fold induction relative to results obtained from albino mice kept for 2 weeks in complete darkness with no exposure to damaging light (0-h). Values are presented as mean ± SEM, *N* = 4; **p < 0.01; ***p < 0.001; ^##^*p* < 0.01 compared to all data points.

We proceeded to test the potential association of ATF3 with the AChE-promoter *in vivo*, using ChIP. Immunoprecipitation was performed with either: (I) A non-relevant normal rabbit IgG, (II) A positive control anti-Histone 3 (H3), or (III) a relevant anti-ATF3 polyclonal antibodies. The precipitated purified DNA was used as a template for real-time PCR reaction with primers annealing at −2.3 kb to −2.1 kb of the mouse AChE-promoter. PCR primers located between +4 bp to +26 bp within the mouse AChE coding region (first exon) served as negative control. PCR primers for H3 served as an additional positive control. Real time PCR results received from the ChIP tested with the non-relevant normal rabbit IgG, did not show any differences between control (0-h light exposure) and the light-stressed albino mice retinas, at any exposure time to the damaging light (Supplementary Figure S1). Similar results, showing no differences between retina samples at any exposure time to damaging light, were received from the ChIP tested with anti-Histone 3 antibody and Histone 3 primers (Figure 5, open squares). However, differences of ~5, 7 and 3-fold increases in ATF3-CRE-binding activity relative to control were found between ChIP results obtained by using anti-ATF3 antibodies and the appropriate primers located within the mouse AChE-promoter (Figure 5, open circles), after 2-, 10- or 24-h of light damage, respectively. Results obtained from the same ChIP samples tested with negative control primers located within the AChE coding region (Figure 5, open inverted triangles) did not show any significant differences compared to control. Our data show that ATF3 directly interacts with AChE promoter *in vivo*, consistent with our *in vitro* observations (see above) and support the hypothesis that ATF3 affects AChE’s expression by modulating the actions of its promoter during stress.

**Figure 6:**
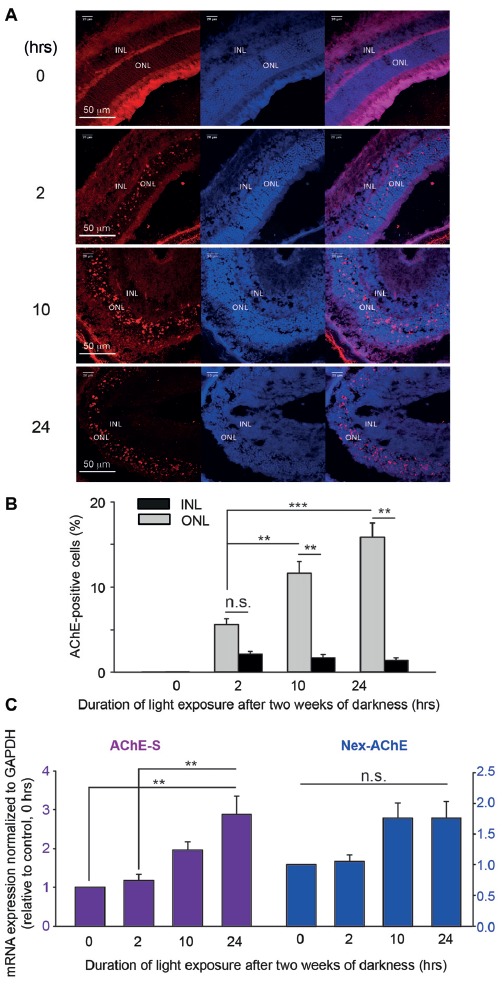
AChE expression after photic-stress in albino mice retina. **(A)** Immunocytochemistry of retinal sections of four albino mice, each exposed to light for different duration, 0- (A1), 2- (A2), 10- (A3) or 24-h (A4). AChE-positive cells (red stain), cell nuclei (blue stain—DAPI) and a merge of the two are shown. **(B)** AChE positive cells, and total number of cells were counted (IMARIS^®^ scientific 3D/4D image processing and analysis *software*) to derive the percentage of AChE positive cells in the ONL and in the INL of each retina, and averaged. Values are mean ± SEM (*N* = 5, one mouse from each group from five independent experiments). Percentage of AChE positive cells in ONL of mice exposed to light for 10- or 24-h, were significantly (**p < 0.01; ***p < 0.001) larger than in the 2 h exposed group of mice. Percentage of AChE positive cells in the ONL were significantly (**p < 0.01) larger compared to INL for 2 and 10-h durations of light exposure. **(C)** Real time PCR was performed on total RNA, extracted from retinas of mice exposed to bright light for 0-, 2-, 10- or 24-h, and tested for the AChE variant induced by light exposure. Testing for the S variant of the C-terminus, and for the extended N terminus are shown in **(C)**. AChE—Ct values were normalized to GAPDH—Ct values. Data are plotted as fold induction relative to the levels of albino mice kept for 2 weeks in complete darkness and not subjected to light exposure. Values are mean ± SEM (*N* = 4, one mouse from each group from four independent experiments); **p < 0.01. n.s., non-significant.

Next step consisted of determining the expression of AChE and ATF3 in the mouse retina, *in vivo*, following photic-stress. Immunohistochemistry for AChE and ATF3 was done on frozen sections of retinas exposed to damaging light. Whereas at time 0 (unperturbed retina) AChE was barely noticeable, the expression of AChE in “blinded”-retinas from albino mice was readily detected after 2-h of exposure to photic-stress and further intensified in retinas undergoing longer light-exposures (Figure 6A). Intriguingly, expression of AChE was almost exclusively detected in the ONL (Figure 6A), reminiscent of the confined localization of apoptotic nuclei (Figure 4). We calculated the percentage of AChE-positive cells in ONL and INL relative to the entire DAPI-stained population of cells in the appropriate nuclear layer. A significant increase was found in the percentage of AChE-positive cells in the ONL of retinas undergoing 10- (11.6% ± 1.4) or 24-h (15.9% ± 1.7) exposure to bright damaging light in comparison to 2-h exposure, as well as compared to positive cells in the INL (Figure 6B gray bars). The percentage of AChE-positive cells in the INL also tended to increase in all three groups, albeit very modestly (by ~2% compared to control) and did not reach statistical significance.

### Identity of the AChE Variant That Drives Apoptosis in Retinas and ATF3 Expression *in Vivo*

To determine the identity of the AChE isoform that is mostly expressed following photic-stress, and likely implicated in promoting photoreceptor apoptosis, we extracted mRNA from “blinded”-retinas and analyzed it using real-time PCR. We, and others, have previsouly used this method to determine the involvement of the N-extended AChE variants in cellular apoptosis following stress (Toiber et al., 2008, 2009; Masha’our et al., 2012). In each real-time-PCR trial, the amplification of each mRNA of different AChE isoforms was normalized to GADPH mRNA and displayed as normalized to the data obtained from retinas of mice that were not exposed to bright light (0-h; see “Materials and Methods” section and Figure 6C). We found gradual and consistent increases in mRNA expression of the AChE-S isoform the longer the retinas were subjected to photic-stress, with retinas subjected to 24-h exposure displaying almost three fold increases (2.9 ± 0.47 fold) in expression when compared to retinas from mice subjected for 0- or 2-h to bright light (Figure 6C, purple bars). To further determine the nature of the N-terminus (normal or extended; Nex) of this AChE-isoform, we used primers to detect the expression of mRNA of the N-extended AChE isoform in “blinded”-retinas. Small, albeit gradual increases, were observed in the expression of the Nex-AChE isoform after 10-h (1.76 ± 0.25 fold expression compared to control) and 24-h (1.76 ± 0.27 fold) following photic-stress (Figure 6C, blue bars). However, these results did not reach significance, leading us to consider the normal (non N-extended)-AChE-S isoform as the dominant isoform that undergoes induction during photic-stress. The AChE-R isoform did not show any changes in expression in the retinas (Supplementary Figure S2). We did not pursue the E-isoform, owing to its exclusive expression in erythrocytes. Together, we conclude from our results that the photic-stress of albino mice causes a robust increase in the normal-AChE-S isoform, *in vivo*. Thereby, this is likely the main isoform detected in nuclei of the photoreceptors located in the ONL (Figures 6A,B).

**Figure 7:**
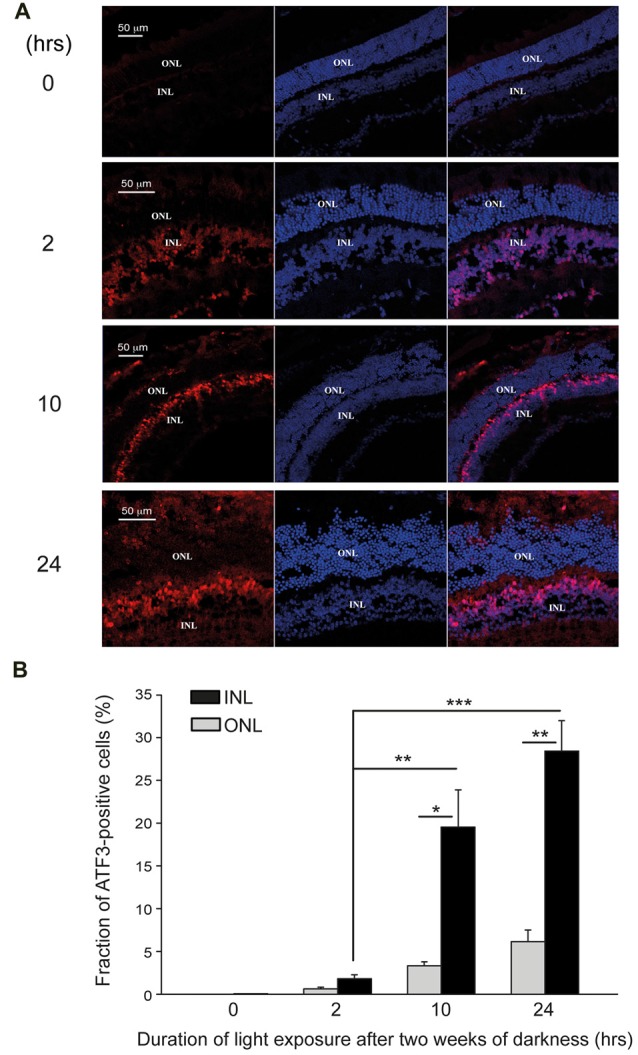
ATF3 is upregulated following photic-stress of albino mice’s retinas. **(A)** Immunocytochemistry of retinal sections of albino mice exposed for 0-, 2-, 10- or 24-h to bright damaging light after 2 weeks in darkness. Cell nuclei (DAPI staining), and ATF3 protein expression are shown (blue and red staining respectively). **(B)** Mean ± SEM, (*N* = 4, one mouse from each group from 4 independent experiments) of the percentage of ATF3 positive cells in the INL is significantly (*p < 0.05; **p < 0.01) larger compared to the corresponding values for the ONL. Fractions of ATF3 positive cells in the INL of mice exposed to light for 10-h or 24-h are significantly (***p < 0.001) larger than in the mice exposed to light for 2-h.

To see the relationship between degenerating retina, AChE and ATF3, we repeated our immunohistochemical analysis, focusing on ATF3. Immunohistochemistry for ATF3 expression in retinas, each belonging to a different group, showed no ATF3 positive nuclei in the retina from the control group (0 h exposure to bright light), and only few in the retina from the mouse exposed for the shortest (2-h of bright light) duration (Figures 7A,B). In contrast, ATF3 positive nuclei were clearly evident in the INL, but not in the ONL, in retinas from mice subjected to 10- or 24-h exposure to bright damaging light (Figures 7A,B). In mice undergoing 2-h exposure to bright light, the INL count was 1.8 ± 0.45% compared to 0.59 ± 0.2% in the ONL. Following longer durations of light exposure, the difference of ATF3 positive cells fraction between INL and ONL was more pronounced; 10-h exposure to light yielded INL—19.6 ± 4.3% and ONL—3.53 ± 0.47%, and following 24-h exposure to light, the percentage of ATF3 positive cells was INL—28.4 ± 3.5% and ONL—6.15 ± 1.25%. Thus, the expression of ATF3 is also enhanced during photic-stress, but the expression is segregated from that of AChE, essentialy localized to the INL of the retina.

## Discussion

Here, we show that ATF3, a transcription factor that is induced by a variety of stress signals including myocardial ischemia (Okamoto et al., 2001), chemical toxicity of the liver (Chen et al., 1996) and renal ischemia-reperfusion (Li et al., 2010), down-regulates the expression of AChE, another stress-induced protein. We hypothesized that, since the promoter of AChE includes the Cyclic AMP-Responsive Element (CRE) consensus sequence, a site that is known to bind CREB/ATF in human AChE-promoter (Choi et al., 2001), then ATF3 and AChE-promoter could potentially interact. Indeed, we show that overexpression of ATF3 down-regulates the expression of AChE-promoter activity (Figure 1). In a different set of experiements, we also demonstrate etoposide-induced expression of AChE in MEF’s devoid of ATF3, but not in MEF stably-expressing ATF3 (Figure 2). Since AChE can promote cellular apoptosis during stress, our observations suggest that ATF3 can protect cells from stress, by limiting the expression of AChE to stay clear of apoptosis. Our hypothesis is supported by a recent study showing that ATF3 attenuates hypoxia-induced neuronal apoptosis by down-regulating the expression of the pro-apoptotic factor carboxyl-terminal modulator protein (CTMP), through binding of the ATF/CREB site in the CTMP-promoter (Huang et al., 2015).

In order to test the effects of ATF3 on AChE, *in vivo*, we chose the mouse model of photic-stress-induced retinal degeneration, which we previously used to show the upregulation of AChE in photoreceptors apoptosis (Kehat et al., 2007). Balb/C albino mice, that are kept in complete darkness for 2 weeks and then exposed to bright damaging light for prolonged durations, display extensive functional damage to the distal retina as assessed by ERG recordings (Figure 3), and extensive apoptosis as assessed by TUNEL staining (Figure 4). Surprisingly, we find for the first time that apoptosis is restricted to the ONL where photoreceptors cells’ nuclei are located (Figure 4), whereas the proximal retina was relatively spared, showing only minor signatures of apoptosis in the inner nuclear layer (INL), and in the ganglion cell layer (Figure 4). Indeed, ChIP assays demonstrate interaction between ATF3 and AChE-promoter in light-damaged retinas but not in control retinas (Figure 5), further confirming our *in vitro* results (Figure 1).

Immunohistochemistry labeling display a strong up-regulation of AChE by photic-stress in the distal retina, the layer occupied by photoreceptors nuclei (Figure 6); with minimal changes in the proximal retina, supporting our previous findings in albino rats (Kehat et al., 2007). Our attempts to determine the identity of the isoform depict the normal AChE-S isoform to be the key mediator of apoptosis (Figure 6C, purple), and not the N-extended type (or at least to a much lesser extent; Figure 6C, blue). These observations, however, diverge from previous reports suggesting the AChE-S of the N-extended kind to play a major pro-apoptogenic role in samples of brain tissue from Alzheimer’s disease patients (Toiber et al., 2008) or in fertilized mouse oocytes (Toiber et al., 2009). These discrepancies may stem from the differences in the methods used for detection of the mRNA (*In Situ* Hybridization [ISH]-there vs. real-time PCR-here), but more likely that these differences stem from the different prepartions used (human samples and oocytes vs. mouse retina). In support of the latter hypothesis are our results with the AChE-R isoform. Whereas in previous studies (Kehat et al., 2007; Masha’our et al., 2012), we find that photic-stress, in albino rats or Y79 retinoblastomas cells, induces strong up-regulation of the AChE-R isoform, here in albino mice we find that the R-isoform remains utterly unchanged (Supplementary Figure S2). Therefore, the combined results from this report, and those presented elsewhere (Toiber et al., 2008, 2009), emphasize the role of the S-isoform in apoptosis, but here we further refine the analysis to conclude that the normal-AChE-S isoform is the major pro-apoptotic mediator in albino mice undergoing photic-stress.

ATF3 expression was also up-regulated by photic-stress in the albino mouse retina (Figure 7), but its localization was segregated from that of AChE. Precisely, AChE’s expression was mainly localized to the ONL (Figure 6), whereas ATF3’s expression was found mainly in the distal border of the INL (Figure 7). Our *in vitro* experiments support the hypothesis that the transcription factor ATF3 down-regulates the expression of AChE, an apoptotic promotor during stress. In the rodent model of light-induced retinal degeneration, damage is restricted to the photoreceptors (ONL) where AChE expression is upregulated during photic-stress. Together, our results suggest that expression of ATF3 in cells in the INL during photic-stress plays a protective role from apoptosis, by down-regulating expression of AChE. We suggest this as a new mechanism exploited by the proximal retina to prevent the expression of AChE; to protect inner retinal neurons during excessive exposure to light and degeneration.

Despite our unique observation that ATF3 is not upregulated in all layers of the retina, it is not completely surprising as ATF3 is not ubiquitously upregulated in all cells. For instance, though many glia cells show upregulation of ATF3 when in the vicinity of injured neurons, not all do so (Hunt et al., 2004). It is suggested that the expression of ATF3 may be selectively and tightly regulated in different cells and tissues to elicit differing responses, such as limiting differentiation and regeneration. Here, we show that ATF3 is exclusively upregulated in the INL but not ONL and suggest this to play a localized role in the protection of the INL by suppressing expression of AChE. In support, it has been suggested that the INL is less damaged by visible light; hypothesized to result from inefficient light absorption by resident proteins or due to the its high glutathione levels serving to block oxidative damage (Winkler, 2008). Indeed, in nocturnal rodents, retinal cell loss is largely restricted to the ONL, as is shown here and elsewhere (Organisciak and Vaughan, 2010). Our results support and extend these observations by providing a possible molecular mechanism behind these elusive observations. The segregated upregulation of ATF3, whether resulting from differences in light intensity, absorption capabilities or ability to cope with oxidative stress (or other) between the layers of the retina, could engender exclusive protection to the INL, but less so in the ONL, where AChE may lead to apoptosis.

In summary, this study sheds light on the molecular mechanism of apoptosis of the retina, *in vivo*. We elucidate the identity of the AChE isoform that is up-regulated during stress (AChE-S) of the retina. We also describe the direct association of ATF3 with the AChE-promoter. Lastly, we propose a novel mechanism by which the retina spares INL neurons, namely by specifically controlling the expression of AChE by ATF3.

## Author Contributions

RHeinrich, SB and IP conceived the study and wrote the manuscript. RHertz and RHeinrich conducted most of the experiments and analyzed the data. EZ and AM assisted with the ERG measurements. IM and LB assisted with the immunohistochemistry preparations.

## Conflict of Interest Statement

The authors declare that the research was conducted in the absence of any commercial or financial relationships that could be construed as a potential conflict of interest.

## Supplementary Material

FIGURE S1 Control IgG. Real time PCR results obtained from the ChIP tested with the non-relevant normal rabbit IgG, between control (0-h light exposure) and the light-stressed albino mice retinas, at varying exposure times to damaging light. Values are presented as mean ± SEM, *N* = 4; n.s., not-significant.

FIGURE S2 The AChE R isoform does not undergo upreguliton in response to photic-stress. Real time PCR was performed on total RNA extracted from retinas of mice, exposed to bright light for 0-, 2-, 10- or 24-h, and tested for the AChE-R variant (see “Materials and Methods” section for primers) induced by varying durations of light exposure. n.s., not-significant.
