# Supplementary figures and images for: ATF3 Regulates the Expression of AChE During Stress
Source: Front Mol Neurosci. 2018 Apr 6;11:88. doi: 10.3389/fnmol.2018.00088 (PMC5897425; doi:10.3389/fnmol.2018.00088)

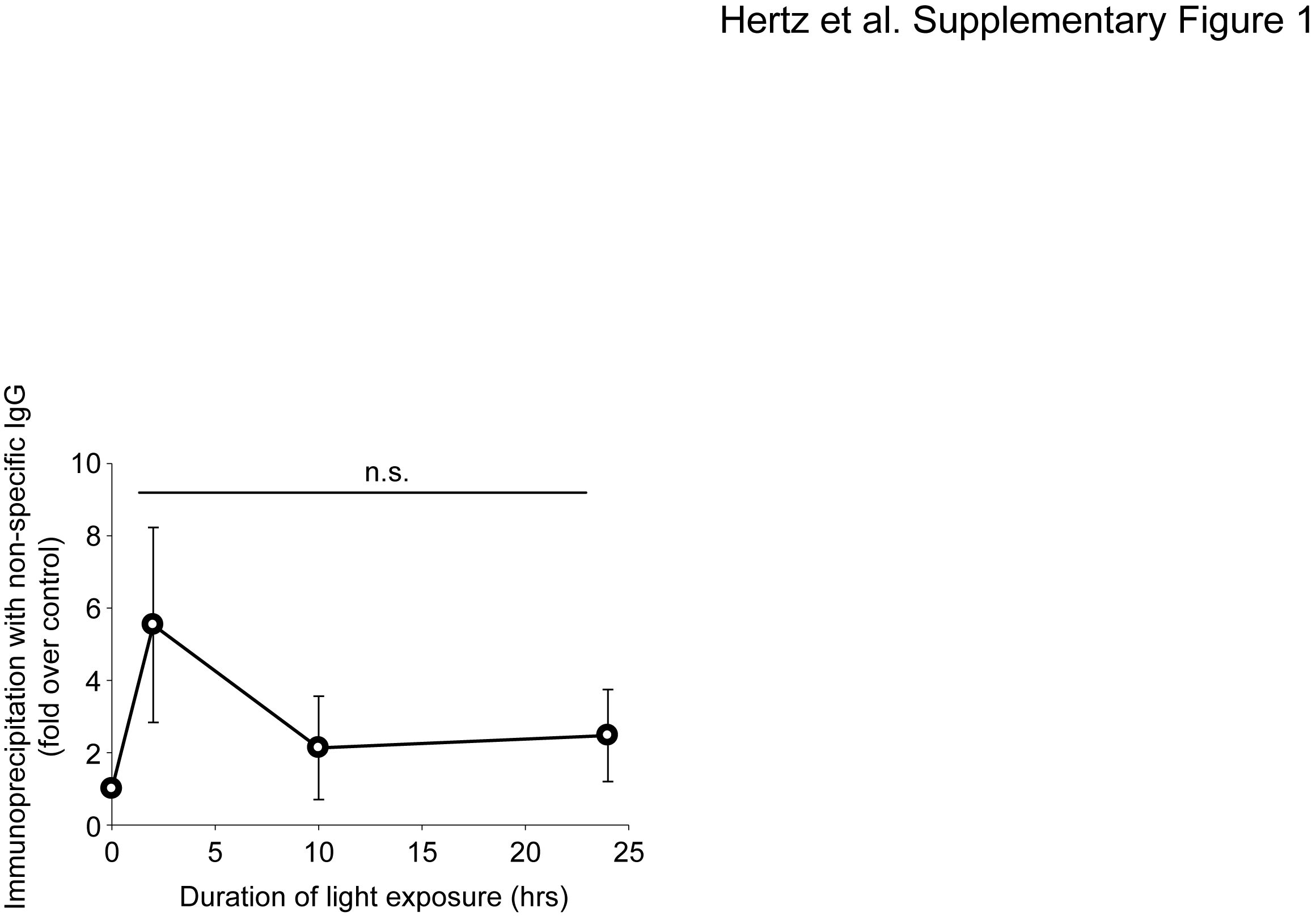

Supplement: FIGURE S1 — Control IgG. Real time PCR results obtained from the ChIP tested with the non-relevant normal rabbit IgG, between control (0-h light exposure) and the light-stressed albino mice retinas, at varying exposure times to damaging light. Values are presented as mean ± SEM, N = 4; n.s., not-significant. [file Image_1.TIF]

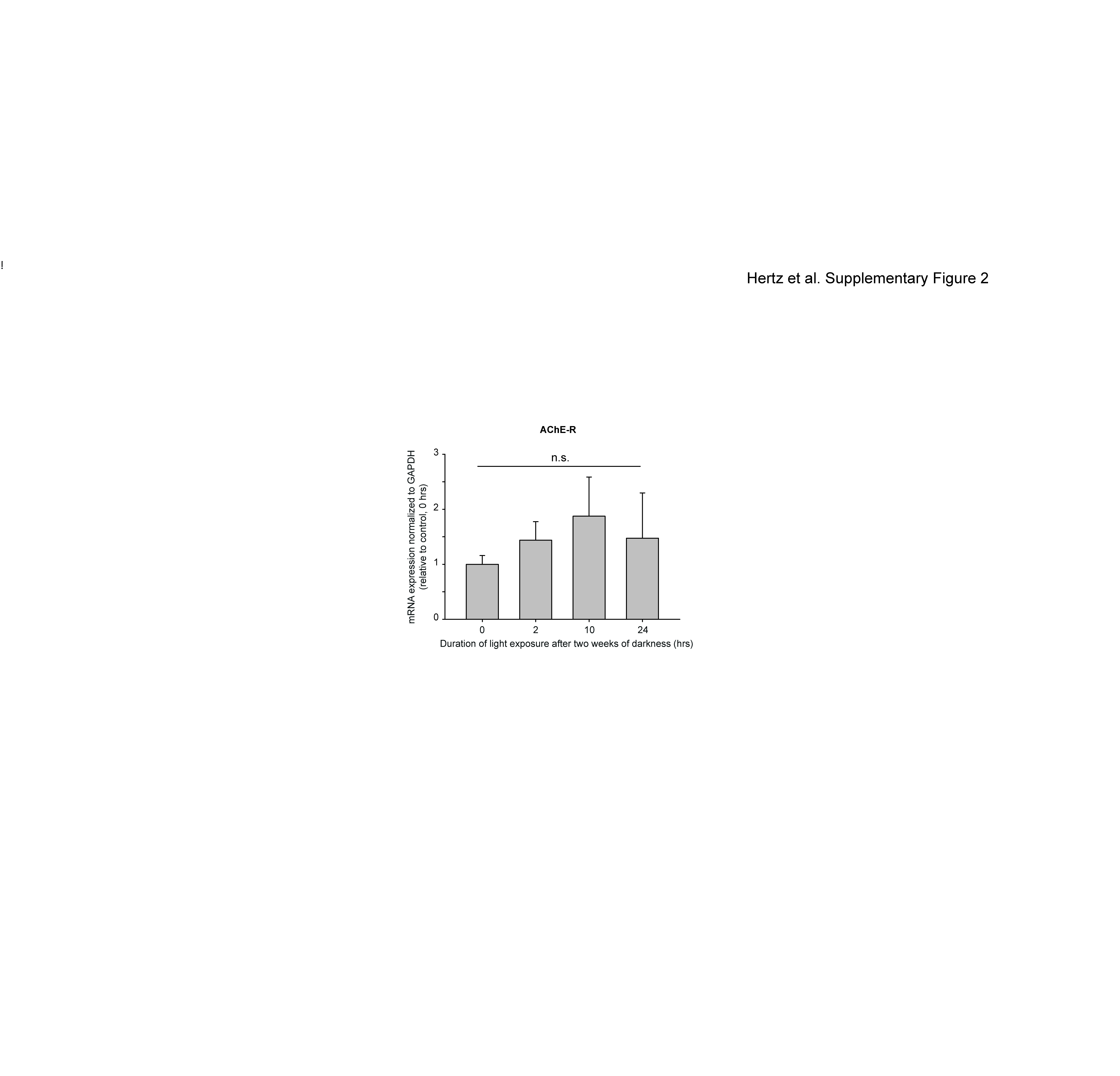

Supplement: FIGURE S2 — The AChE R isoform does not undergo upreguliton in response to photic-stress. Real time PCR was performed on total RNA extracted from retinas of mice, exposed to bright light for 0-, 2-, 10- or 24-h, and tested for the AChE-R variant (see “Materials and Methods” section for primers) induced by varying durations of light exposure. n.s., not-significant. [file Image_2.TIF]
